# Case Report of Painless Obstructive Jaundice: A Rare First Presentation of Small-Cell Lung Cancer

**DOI:** 10.7759/cureus.35561

**Published:** 2023-02-27

**Authors:** Prabasha Weeraddana, Mikhail Dmitriev, Teena Thomas, Wenli Gao, Niwanthi Weerasooriya, Fnu Sandeep

**Affiliations:** 1 Internal Medicine, Danbury Hospital, Danbury, USA; 2 Oncology, Danbury Hospital, Danbury, USA; 3 Pathology, Danbury Hospital, Danbury, USA

**Keywords:** chemotherapy, new-onset jaundice, liver metastasis, intrahepatic cholestasis, small-cell lung carcinoma, painless obstructive jaundice

## Abstract

Small-cell lung cancer (SCLC) is a very aggressive type of lung cancer that is of neuroendocrine origin. Because of the high levels of circulating tumor cells, it has a very high rate of metastasis. Obstructive jaundice as the initial manifestation of small cell lung carcinoma is rare. Most of the cases are due to extrahepatic cholestasis by biliary duct obstruction. The biliary duct obstruction may be secondary to metastasis to lymph nodes or pancreatic head metastasis. Obstructive jaundice secondary to intrahepatic cholestasis is even rarer. A 75-year-old male presented to the emergency department (ED) with a complaint of new-onset painless jaundice that his dentist incidentally detected. Examination revealed a mass in the right upper quadrant (RUQ) of the abdomen. Computed tomography (CT) angiography of the abdomen, pancreas, and pelvis shows innumerable hepatic hypodensities highly suspicious for metastatic disease. However, there was no extrahepatic dilatation or pancreatic mass. He was diagnosed with diffuse metastasis of small cell lung carcinoma (SCLC) by needle biopsy of the liver. He developed acute kidney injury and liver damage and thus compromised chemotherapy for SCLC. Later, the patient chose comfort care and passed away the next day. To our knowledge, this is the second reported case of SCLC initially presenting as obstructive jaundice secondary intrahepatic cholestasis by diffuse liver metastases.

## Introduction

About 15% of all lung carcinomas are small cell lung cancers (SCLC), the most dangerous type of lung cancer [1]. Its proliferative aggressiveness and high rate of metastasis contribute to its high mortality rates. The concentration of circulating tumor cells (CTCs) in SCLC is among the highest of any solid tumors, mirroring its high propensity for metastatic spread [2]. It is strongly associated with cigarette smoking [3]. In most cases, patients arrive with short-lived symptoms and frequently (60%-65%) have the metastatic disease, which eliminates the ideal circumstances for examining the development of tumorigenesis and gene alterations. SCLC typically has a good response to chemotherapy and radiation. Patients with SCLC typically present with respiratory symptoms such as cough, dyspnea (labored breathing), or hemoptysis (coughing up blood), with imaging revealing a central lung mass and frequent lymph node involvement. The contralateral lung, the brain, the liver, the adrenal glands, and the bone are the most typical locations for metastasis of SCLC. Jaundice usually occurs as a late manifestation of SCLC due to widespread metastasis, particularly to the liver. Here we described a case of SCLC first presented as painless obstructive jaundice secondary to intrahepatic cholestasis.

## Case presentation

This is a 75-year-old caucasian male who presented to the ED with new-onset painless jaundice after being referred by his primary care physician (PCP). The patient recently visited his dentist for a regular checkup, and at that time, he was noted to have jaundice and was asked to have a follow-up visit with his PCP. His PCP ordered lab work, which revealed elevated total bilirubin and liver function test (LFT). He noticed that his urine had been darker than usual but denied pale stools. He denied any fever, chills, abdominal pain or recent weight loss. On admission, he appeared jaundiced, and the sclera was icteric. Abdominal examination revealed a mildly distended abdomen with a firm mass in the right upper quadrant of the abdomen. The abdomen was soft, and there was no significant tenderness or guarding. Pitting edema was noted up to the mid-leg level of bilateral legs.

The patient had coronary artery bypass graft surgery (CABG) about 15 years ago and a cholecystectomy around 13 years ago. His past medical history was also significant for hypertension and hyperlipidemia. He was a current smoker with a 60-pack smoking history per year. He also revealed that he drank alcoholic beverages once or twice per month. His family history was significant for liver cancer (his brother died from liver cancer).

On admission, lab work showed a leukocyte count of 18 × 109/L, indicating leukocytosis (with a predominance of neutrophils), hemoglobin of 11.6 grams per deciliter, a platelet count of 190 × 103 platelets per microliter, a prothrombin time (PT) of 15.7 seconds, and an international normalized ratio (INR) of 1.2. Total bilirubin was 13.3 mg/dL, direct bilirubin was 10.5 mg/dL, alkaline phosphatase was 500 IU/L,gamma-glutamyl transferase (GGT) was 2000 U/L, alanine transaminase (ALT) was 140 U/L, aspartate transaminase (AST) was 200 U/L, amylase was 92 U/L, and lipase was 105 U/L. Urinalysis is positive for bilirubin and protein (1+). His creatinine level was 1.72 mg/dL (significantly higher than his previous lab result of 0.95 mg/dL around two years ago), and his blood urea nitrogen (BUN) level was 26 mg/dL. Furthermore, his lactic acid levels were significantly high at 4.4 mmol/L (indicating lactic acidosis) and improved to 2.9 mmol/L after intravenous (IV) fluid.

**Figure 1:**
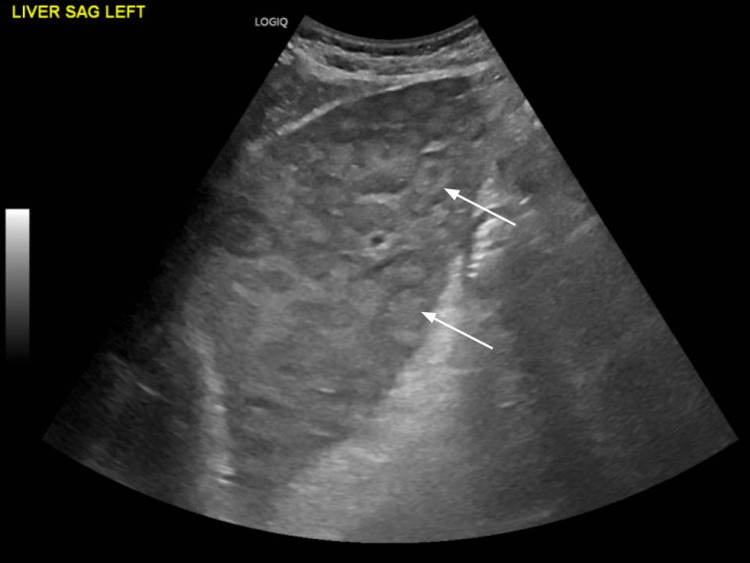
The ultrasound scan (US) of the abdomen revealed an enlarged liver, infiltrated with multiple hyperechoic rounded masses concerning the extensive metastatic disease

**Figure 2:**
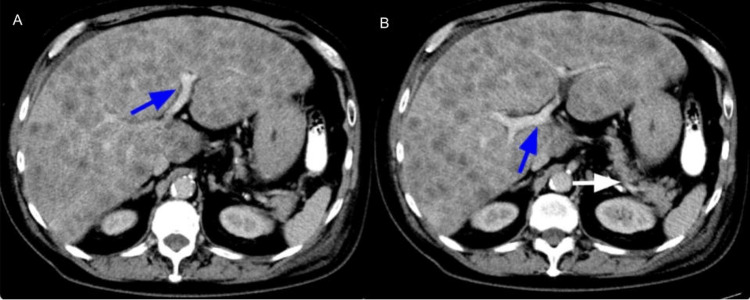
CT angiography of the abdomen, pelvis and pancreatic mass protocol showed no intrahepatic (blue arrow in pictures A and B) or extrahepatic biliary dilatation/mass (white arrow in B picture).

**Figure 3:**
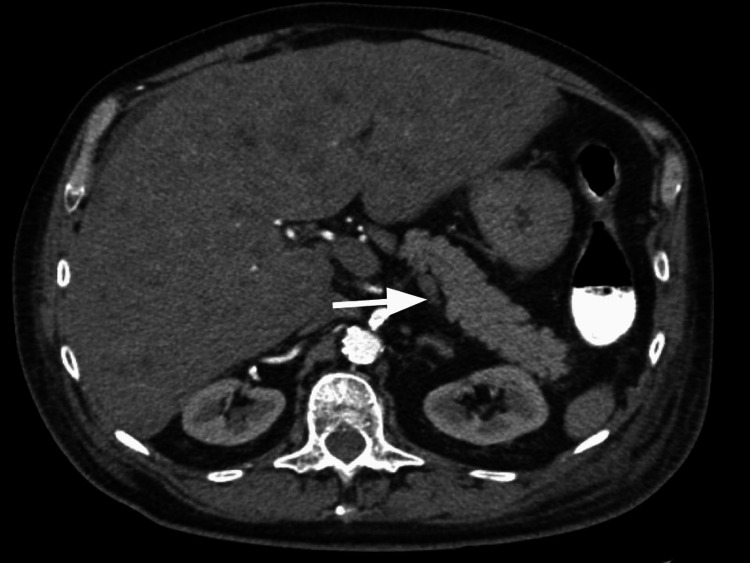
CT angiography of the abdomen, pelvis and pancreatic mass protocol showed no evidence of pancreatic mass.

**Figure 4:**
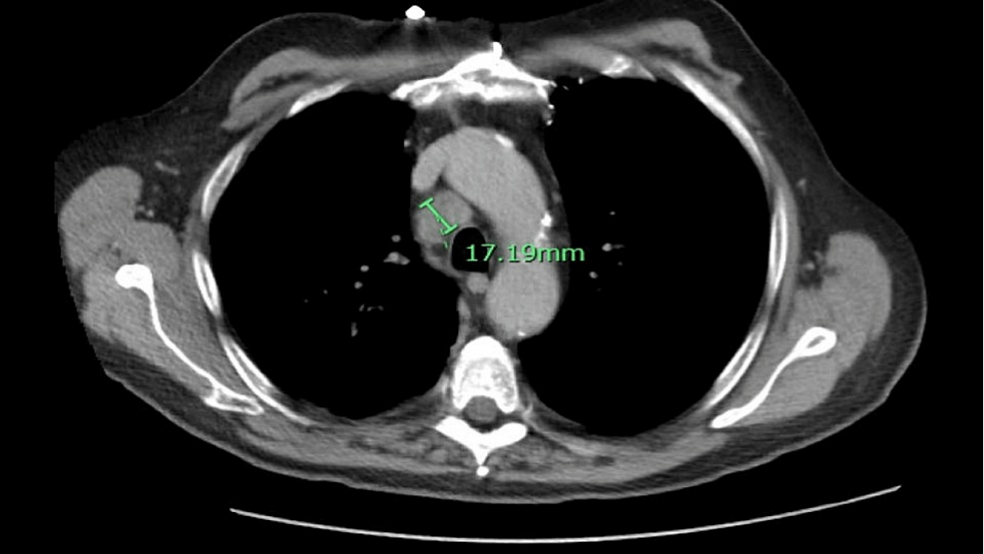
CT angiography of the abdomen, pelvis and pancreatic mass protocol showed a 1.7 cm pretracheal lymph node.

**Figure 5:**
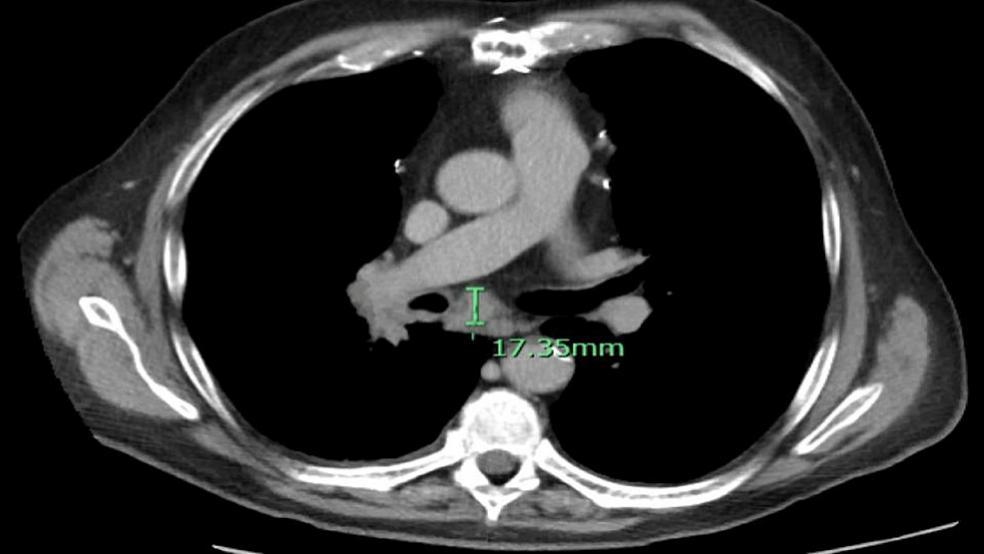
CT angiography of the abdomen, pelvis and pancreatic mass protocol shows subcarinal lymphadenopathy (1.7 cm).

**Figure 6:**
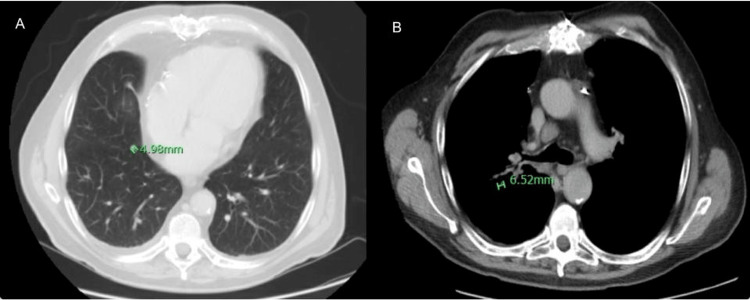
CT angiography of the abdomen, pelvis and pancreatic mass protocol shows about 0.6 cm nodule in the medial right lower lobe (A) and an 0.7 cm nodule in the posterior right perihilar region (B).

**Figure 7:**
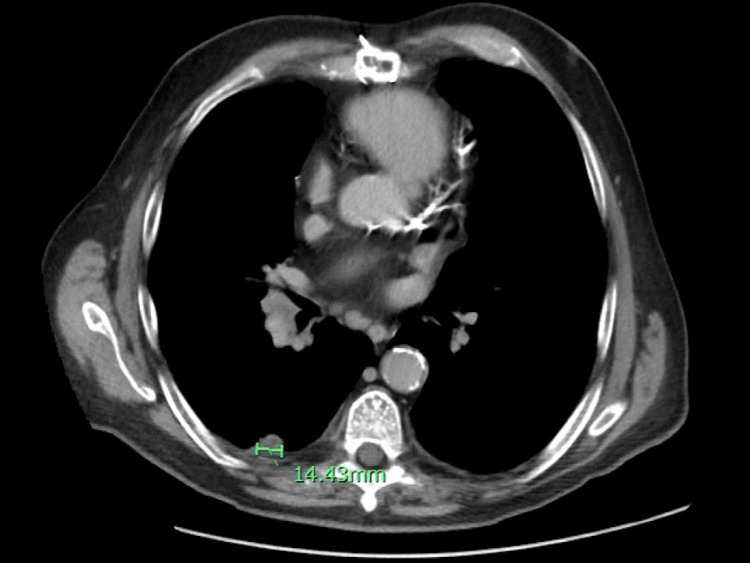
CT angiography of the abdomen, pelvis and pancreatic mass protocol shows a 1.4 cm pleural-based mass posteriorly in the superior segment of the right lower lobe.

The ultrasound scan (US) of the abdomen revealed an enlarged liver, measuring 24 cm, infiltrated with multiple hyperechoic rounded masses concerning the extensive metastatic disease (Figure 1). A follow-up CT angiography of the abdomen, pelvis and pancreatic mass protocol was done, which showed no intrahepatic or extrahepatic biliary dilatation (Figure 2). The spleen was normal. No evidence of pancreatic mass (Figure 3). There were innumerable hepatic hypodensities concerning the metastatic disease. It further revealed pre-tracheal, subcarinal, and right hilar lymphadenopathy (Figure 4-6). There was a pleural-based mass measuring 1.4 cm in the superior segment of the right lower lobe of the lung (Figure 7). This raised the concern of a primary lung malignancy with metastatic disease. There was no significant retroperitoneal or pelvic lymphadenopathy. There was no evidence of anatomically obstructive disease from extrinsic compression of the biliary tree due to masses or lesions.

**Figure 8:**
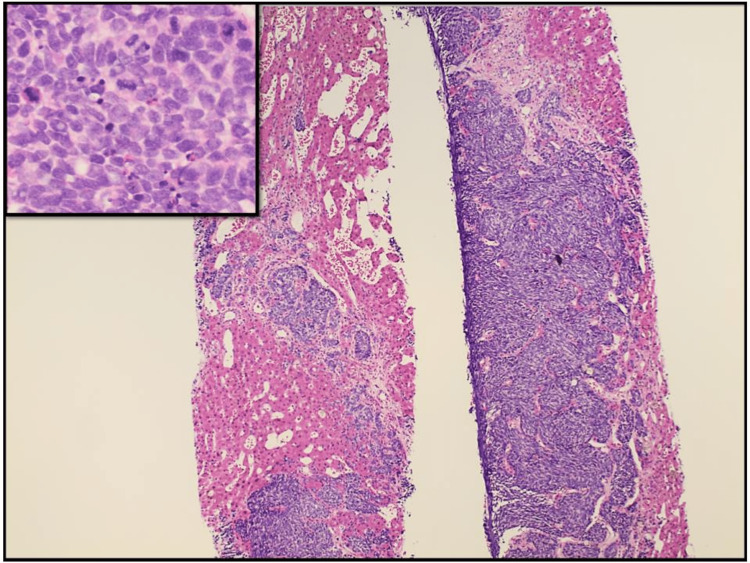
Histology of liver lesion (H&E/Hematoxylin and eosin stain x 200) shows tumor cells that have round/oval blue cells with minimal cytoplasm, finely dispersed chromatin, no distinct nucleoli, or molding, and with high mitotic rate.

**Figure 9:**
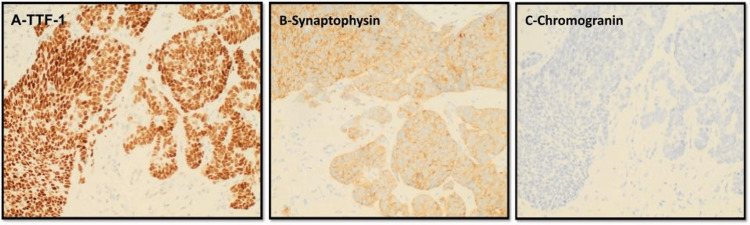
Immunohistochemical staining of biopsy of liver lesions showed that the tumor cells were positive for thyroid transcription factor-1 /TTF-1 (A) and strongly positive for synaptophysin (B) while being negative for chromogranin (C).

The patient underwent an IR-guided needle biopsy of the liver. The pathology report showed metastatic small-cell carcinoma (Figure 8). Immunohistochemical staining showed that the tumor cells were positive for thyroid transcription factor-1 (TTF-1) and strongly positive for synaptophysin while being negative for chromogranin (Figure 9). This staining pattern supported the diagnosis. His hospital course was complicated by the development of acute kidney injury (AKI) with worsening serum creatinine at 2.34 and BUN 48. Due to the aggressive nature of small cell cancer, the patient developed liver failure.

Unfortunately, we could not find any chemotherapy deemed safe because of his liver failure, which led to bilirubin levels up to 14 mg/dL and renal failure. The usual chemotherapy for metastatic small cell cancer consists of carboplatin and etoposide, carboplatin and irinotecan, or carboplatin and paclitaxel. This regimen could not have been given to the patient due to hepatic and renal failure. Thus, carboplatin had to have its dose reduced, and due to his kidney failure, cisplatin was contraindicated. Without any treatment, his metastatic small cell carcinoma was lethal. Even with treatment, the chance of the patient responding to it and tolerating chemo was very low, but at least there was a small chance he could have survived the chemotherapy. The patient chose to do not to resuscitate (DNR) and do not intubate (DNI) but requested chemotherapy to be started. He was then subjected to a non-contrast CT brain and head scan, which revealed no intracranial mass. He underwent chemotherapy with a renal-adjusted dose of carboplatin and a 50% reduced dose of etoposide. Due to the high risk of tumor lysis syndrome, rasburicase was given before chemotherapy. The patient tolerated the first chemotherapy dose, and the kidney function improved. There was also a mild improvement in alkaline phosphatase, ALT, and AST levels (ALP: 364 U/L; ALT: 196 U/L; AST: 234 U/L). But his bilirubin continues to rise. The day-to-day blood workup is shown in Table 1. 

**Table 1:** Day-to-day blood workup (day 0 being the day of admission) ALP: alkaline phosphatase, ALT: alanine aminotransferase, AST: aspartate aminotransferase, BUN: blood urea nitrogen

Test Name	Day 0	Day 1	Day 2	Day 5	Day 6	Day 7	Day 8	Day 9	Day 10	Day 11	Day 12	Day 13	Day 14
Creatinine(mg/dL)	1.72	1.49	1.46	1.54	2.10	2.34	2.11	1.83	1.64	1.59	1.49	1.44	1.48
BUN	26	26	25	22	35	48	50	43	38	38	36	35	32
BILIRUBIN TOTAL (mg/dL)	13.3	13.2	13.4	13.8	14.8	14.3	14.4	15.8	18.3	17.8	20.1	22.8	21.7
BILIRUBIN DIRECT (mg/dL)	10.5	10.9	11.4	10.9	12.3	10.7	11.6	12.5	14.7	14	15.7	17.7	16.6
ALP (IU/L)	557	479	458	442	407	410	344	355	364	351	371	387	381
ALT (U/L)	139	122	125	125	253	340	278	252	196	163	155	139	113
AST (U/L)	209	190	207	459	568	416	374	234	216	226	208	162	132

On hospital admission day 12^th^, the patient was noted to have bilateral frank ecchymosis. Therefore, the CT abdomen and pelvis without contrast was taken, which revealed moderate abdominal and pelvic free fluid with dependent high-density material within the pelvic fluid that could represent hemorrhagic or proteinaceous content; however, the findings were limited by the lack of contrast. On the 13^th^ day of hospitalization, the patient was noted to be more dyspneic and lethargic; he was noted to have a low-grade fever of 100.2°F, was tachycardic and hypotensive, and had signs of sepsis. He was started on cefepime; however, the patient expressed a desire to be made comfortable and to no longer pursue invasive procedures. He was transitioned to a "comfort measures only" status and passed away the next day.

## Discussion

Small cell lung cancer (SCLC), the most aggressive type of lung cancer, makes up about 15% of all lung carcinomas [1]. It is strongly associated with cigarette smoking [3]. It has high mortality rates because of its characteristically aggressive proliferation and high rate of metastasis [4]. Imaging typically reveals a central lung mass and frequent lymph node involvement, and they frequently present with respiratory symptoms like cough, dyspnea (labored breathing), or hemoptysis (coughing up blood). SCLC differs from non-small cell lung cancer on the histological basis that establishes morphological differences between the two. In light microscopy, SCLC typically has a higher nuclear/cytoplasmic ratio, finely granular nuclear chromatin, no nucleoli, and a fusiform shape [5]. Various mechanisms result in small-cell lung cancer. Approximately 75% to 90% of SCLC patients have p53 inactivation [6]. Pulmonary neuroendocrine cells (PNECs) occurring in proximal airways as solitary cells or in intralobar airways as neuroepithelial bodies (NEBs) (clusters) have a role in the development of SCLC [7]. Following pathological or external injury, PNEC hyperplasia results, which progresses to SCLC. 

The majority of reported cases of obstructive jaundice due to cancer metastasis are due to extrahepatic metastasis. Obstructive jaundice due to pancreatic metastasis has been reported in various studies [8,9]. Incidence of obstructive jaundice due to intrahepatic cholestasis by diffuse hepatic metastasis is extremely rare. Due to the lack of specific symptoms and rapid tumor growth, early detection of SCLC is difficult.

Chemotherapy is still the go-to treatment for small cell lung cancer (SCLC) [10]. Etoposide or irinotecan, in combination with platinum, makes up the first-line standard chemotherapy [11]. For patients with extensive-stage SCLC treated with frontline chemotherapy, the median overall survival (OS) is only about 10 months [12]. Minimal therapeutic and clinical advancement has been made over the past 30 years, earning SCLC the moniker "recalcitrant cancer” [13]. The use of single-agent regimens of standard cytotoxic agents like paclitaxel as a second-line treatment choice for small-cell lung cancer is currently being investigated.

The patient we present had obstructive jaundice due to hepatic metastasis of SCLC. The patient was asymptomatic, and the diagnosis of jaundice was made accidentally when he presented to his dentist for a routine checkup. Previously, only one such case presentation has been reported, in which the patient presented with obstructive jaundice due to diffuse metastatic disease that developed because of small-cell lung cancer [14]. 

## Conclusions

To the best of our knowledge, this is the second reported case of obstructive jaundice due to intrahepatic cholestasis caused by diffuse hepatic metastasis of small-cell lung cancer. Liver metastasis is a poor prognosis indicator of small cell carcinoma, posing a challenge to find the best suitable chemotherapy regimen in case of elevated bilirubin levels. It is vital to screen patients at high risk for lung carcinoma, such as long-term smoking with CT chest, for early diagnosis and to prevent poor outcomes. 
